# A delayed post-operative diaphragmatic hernia with hemothorax due to a strangulated stomach

**DOI:** 10.1093/jscr/rjaa515

**Published:** 2020-12-28

**Authors:** Rama Al-Saqqa, Rami Sabouni, Lana Jarad, Nizar Abbas

**Affiliations:** Faculty of Medicine, Damascus University, Damascus, Syria; Faculty of Medicine, Damascus University, Damascus, Syria; Faculty of Medicine, Damascus University, Damascus, Syria; Department of Thoracic Surgery, Al-Assad University Hospital, Damascus, Syria

**Keywords:** traumatic diaphragm hernias, echinococcus, hernia, Iatrogenic

## Abstract

Traumatic diaphragmatic hernias (TDHs) are uncommon, and they mostly occur following blunt or penetrating traumatic injury and rarely as a complication of Iatrogenic procedure. The management of TDHs is through surgical repair. In this article, we present the case of a diaphragmatic herniation presenting 1 year after thoracic surgery in a 16-year-old male. The patient presented with gastrointestinal obstruction symptoms and later developed a hypovolemic shock due to stomach bleeding. Immediate exploratory thoracotomy was performed, and the patient reached a full recovery afterward.

## INTRODUCTION

Traumatic diaphragmatic hernias (TDHs) are uncommon but life-threatening, and they remain a diagnostic and therapeutic challenge [1]. TDHs have an overall mortality rate of up to 31% in recent series [2]. They most commonly occur following blunt or penetrating traumatic injury, which results in the rupture of the diaphragm with herniation of abdominal contents [3]. Such trauma is rarely caused by a surgical intervention [4]. The herniation may present months or years later [5].

**Figure 1:**
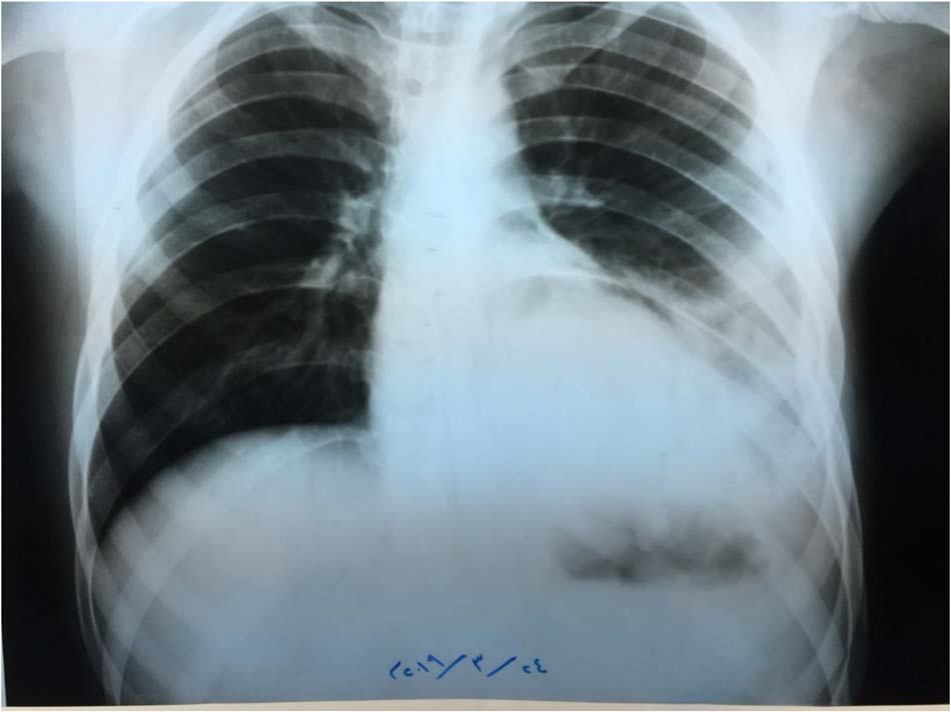
Chest X-ray.

In this case, we report a patient suffering from gastrointestinal symptoms caused by a delayed TDH.

**Figure 2:**
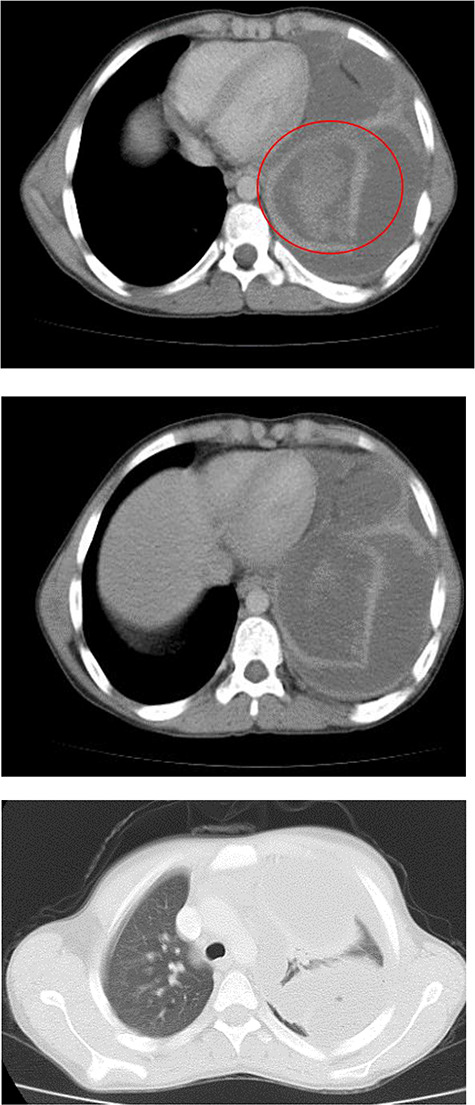
Chest CT.

## CASE PRESENTATION

A 16-year-old male presented to the outpatient clinic complaining of epigastric pain that radiates to the left hypochondrium accompanied by severe vomiting immediately after eating or drinking. No other associated symptoms existed; his medical history was clear of any significant complaints. He denied any thoracic or abdominal trauma, but his surgical history included the resection of a hydatid cyst in the lower zone of the left lung through a thoracic approach 1 year previous to the current complaint. No further information about the surgery was available.

On examination, we found a decrease in respiratory sounds in the lower zone of the left lung, with the absence of inflammatory signs. The remaining of the physical examination and lab test results were all within normal limits.

Chest X-ray (Fig. 1) showed an increased density in the left lower zone of the lung. Forty-eight hours later, the patient arrived at the ER along with the computed tomography (CT) scan (Fig. 2). He was in a very poor general condition with pallor, severe dyspnea and clouding of consciousness. Physical examination showed the absence of the left lung sounds, shifted apex beat of the heart to the right and a thready pulse and a blood pressure of about 80/40 mmHg.

**Figure 3:**
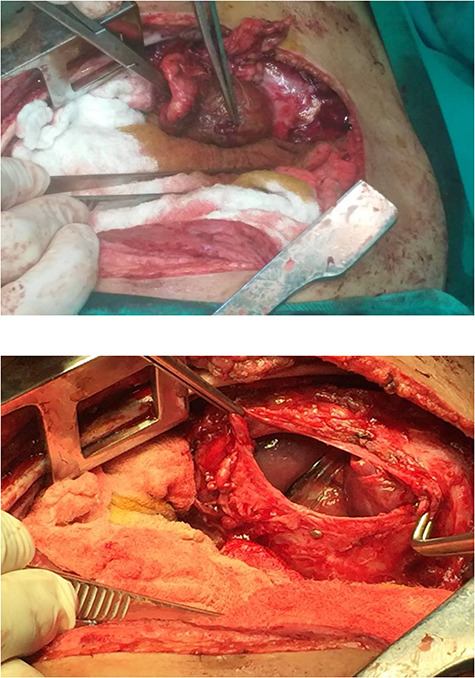
Surgical repair of the diaphragm.

**Figure 4:**
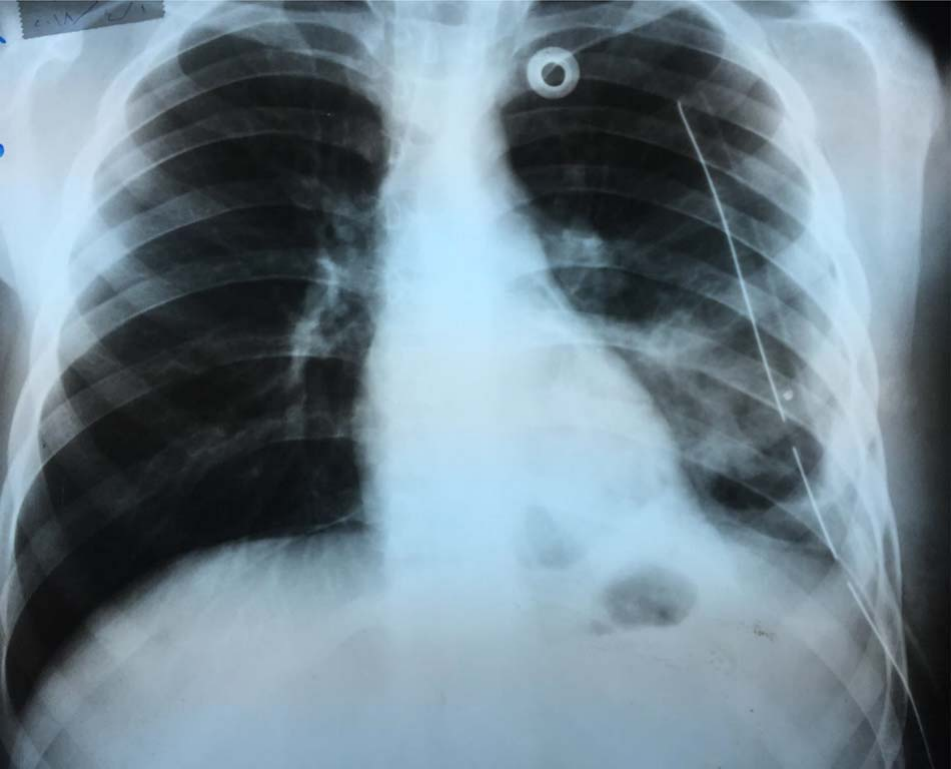
Chest X-ray 24 h after surgery.

A catheter was placed and fluid infusion began, and emergency thoracentesis showed fresh blood. Immediate exploratory thoracotomy was performed (Fig. 3). Three liters of blood were extracted, and the pleural adhesions were liberated. A diaphragmatic hernia was found, with an ischemic strangulated and perforated stomach. The stomach was freed, found vital, its perforation was repaired and it was returned to the abdominal cavity. The opening in the diaphragm was sutured and supported with the omentum. The lung was in good shape.

An X-ray image (Fig. 4) was done 24 h after the operation, and it showed good improvement.

## DISCUSSION

Diaphragmatic hernias are either congenital or secondary due to trauma [6]. TDHs are rare and occur more often after blunt trauma [6].

Approximately, 0.8–3.6% of thoracoabdominal trauma cases include diaphragmatic rupture [7], with a low incidence of herniation afterward [7].

Latrogenic diaphragmatic hernias are a rare complication of abdominal surgery [4], and they most commonly occur after liver transplants and liver resection [8].

In our case, the trauma was probably Iatrogenic, the diaphragm was most likely injured by the adjacent surgical procedure and the herniation occurred within a year.

TDHs presentation varies significantly; it can be asymptomatic and might also have respiratory, abdominal or cardiac symptoms or even failure [9]. Complications have included gastrointestinal obstruction, gastrointestinal strangulation, respiratory failure and cardiac tamponade [8].

In our case, the patient suffered from upper gastrointestinal obstruction symptoms but was only admitted after the complication had occurred, and he presented with hypovolemic shock due to stomach bleeding which caused a hemothorax.

TDH surgical repair involves diaphragm closure through an abdominal or thoracic approach [4]. In our case, thoracotomy was performed.

In conclusion, TDHs might be a differential diagnosis in patients complaining of upper gastrointestinal obstruction symptoms with a history of thoracic surgery. When the diagnosis of TDH is confirmed, immediate intervention might prevent dangerous complications.

## CONFLICT OF INTEREST STATEMENT

None declared.

## FUNDING

None.
